# *MiR-144* inhibits growth and metastasis in colon cancer by down-regulating SMAD4

**DOI:** 10.1042/BSR20181895

**Published:** 2019-03-01

**Authors:** Shihou Sheng, Lin Xie, Yuanyu Wu, Meng Ding, Tao Zhang, Xu Wang

**Affiliations:** 1 Department of Gastrointestinal Surgery, China-Japan Union Hospital of Jilin University, Changchun, Jilin, P.R. China; 2 School of Public Health, Jilin University, Changchun, Jilin, P.R. China; 3 Department of Endoscopy Center, China-Japan Union Hospital of Jilin University, Changchun, Jilin, P.R. China; 4 Department of Colorectal and Anal Surgery, the First Hospital of Jilin University, No.71 Xinmin Road, Changchun, Jilin 130000, P.R. China

**Keywords:** colon cancer, invasion, microRNA144, proliferation, SMAD4

## Abstract

MicroRNAs (MiRs) are thought to display regulator action in tumor suppression and oncogenesis. *miR-144* plays an important role in the development of various cancers, such as colorectal cancer, breast cancer, and lung cancer, by targetting different molecules potentially involved in many signaling pathways. SMAD4 is a common signaling during tumor progression, and it can inhibit cell proliferation and promote cell motility in most epithelial cells. The present study focused on the effect of *miR-144* and SMAD4 on colon cancer in order to find the novel gene therapy target for the treatment of colon cancer. Quantitative real-time polymerase chain reaction was used to assess the expression level of *miR-144* in colon cancer tissues and SW620 cells. MTT assay, scratch test, and transwell assay were used to evaluate cell proliferation, migration, and invasion, respectively. Moreover, luciferase assays were utilized to identify the predictive effect of *miR-144* on SMAD4. Western blotting was performed to determine the relative expression of protein related to SMAD4. We found *miR-144* level was significantly lower in colon cancer tissues and SW620 cells. Moreover, SMAD4 level, both in mRNA and protein, was obviously elevated in colon cancer tissues. Further, *miR-144* mimics treatment inhibited cells proliferation, invasion, and migration. Fluorescence intensity of *miR-144* mimics group in wild type cells was decreased. *MiR-144* mimics repressed the SMAD4 expression both in mRNA and protein. These findings about *miR-144*/SMAD4 pair provide a novel therapeutic method for colon cancer patients.

## Introduction

Colon cancer is known to be the third most common type of cancer in the world and the fourth leading cause of cancer mortality [1,2]. Although there are many treatment methods, such as surgery, surgery combined with chemotherapy and radiotherapy, the median survival rate of colon cancer is still poor [3,4]. The differential expression of microRNAs (miRs) was first reported in relation to colon cancer in 2003 [5], which is essential for understanding the molecular mechanisms of colon cancer. MiRs are closely related to various biological processes, such as cell proliferation, cell cycle progression, apoptosis, motility, and tumorigenesis by directly mediating the expression level of its target gene [6]. In recent years, due to their control of key oncogenes and tumor suppressor gene expression [7], miRNA has been shown to play a key role in cancers.

MiRs are short non-coding RNAs with extensive gene regulatory activity levels after transcription. MiR binds to several proteins in the RNA silencing complex, causing mRNA degradation or translation inhibition, or both processes [8]. MiRs are proposed to show tumor suppressor and oncogene regulator, and develop complex patterns of tissue-specific expression that can affect cancer cell proliferation [9] and metastasis [10], and may define the cancer stem cell phenotype. Originally, *miR-144* was considered to be an erythroid-specific miRNA, which was necessary for survival and maturation of subsequent erythroid lineage [11]. *MiR-144* plays an important role in the development of various cancers, such as colorectal cancer, [9] breast cancer [12], and lung cancer [13] by targetting different molecules of many signaling pathways. SMAD4, identified as a Co-Smad of the Smad family, is a common mediator for transforming growth factor-β signaling pathways [14,15]. As a common signaling during tumor progression, it can inhibit cell proliferation and promote cell motility in most epithelial cells, thus partly can affect sensitivity to clinical therapy [1,16,17].

In this work, carcinoma and matched paracancer tissues in 80 patients were collected for assessing the expression of *miR-144*. SMAD4 level in these samples was also examined. *In vitro*, cell proliferation, invasion, and migration capabilities were tested to observe the effect of *miR-144* on colon cell lines SW620. Moreover, the predictive effects of *miR-144* on SMAD4 were detected using luciferase assays, quantitative real-time polymerase chain reaction (qRT-PCR), and Western blotting. Collectively, we indicated the importance of *miR-144* as a promising gene therapy target to treat colon cancer, demonstrating that *miR-144* is worthy of further investigation.

## Materials and methods

### Tissue samples

Eighty of colon cancer tissues and paired para-carcinoma tissues were obtained through surgical resection after the patients agreed in the hospital between 2009 and 2013. Clinicopathological data were all recorded, including age, sex, tumor location, and histological differentiation. Tissue samples were snapfrozen in liquid nitrogen and stored at −80°C. The colon cancer patients had not received adjuvant therapy (e.g., chemotherapy and radiotherapy) before tissue sampling. This research was approved by the Ethics Committee of China-Japan Union Hospital of Jilin University and written informed consent was provided to all the patients. The present study was conducted in accordance with the Declaration of Helsinki and written informed consent was obtained from the participant.

### Cell culture

Human colon cancer cell lines SW620 and normal intestinal epithelial cells were purchased from the American Type Culture Collection. Cells were incubated in RPMI 1640 medium (HyClone, South Logan, UT, U.S.A.) with 10% heat-inactivated fetal bovine serum (Gibco, Carlsbad, CA, U.S.A.) in a humidified incubator containing 5% CO_2_ at 37°C. The normal colonic epithelial cells were purchased as negative control (NC).

### qRT-PCR

Total RNAs of cells and tissues were extracted by using 1.0 ml TRIzol (Invitrogen, Carlsbad, CA, U.S.A.), according to the manufacturer’s protocol. The ratio measure of optical density (OD) 260/280 for RNA extraction was between 1.8 and 2.0. Synthesis of cDNA was carried out using PrimeScript™ RT Reagent Kit (Takara, Dalian, China). QRT-PCR was performed by SYBR Premix Ex Taq™ Kit (Takara) on QuantStudio™ Real-Time PCR system (Applied Biosystems, Foster City, CA, U.S.A.) following the manufacturer’s protocol. The parameters were as follows: hot start at 95°C for 10 min; followed by 35 cycles of 95°C for 30 s, 60°C for 30 s, and 72°C for 30 s; then extended at 72°C for 10 min. The primer sequences were as follow: *miR-144*, F: ACACTCCAGCTGGGTACAGTATAGATGATGTA, R: CTCAACTGGTGTCGTGGA. SMAD4, F: 5′-AGTCCCTGGATCACCGACAG-3′; R: 5′-GTTTCTTGCCTCTT-GGTTGCT-3′. RNU6 snRNA, F: 5′-CTCGCTTCGGCAGCACA-3′, R: 5′-AACGCTTCACGAATTTGCGT-3′. GAPDH: F: 5′-GATTTGGTCGTATTGGGCGC-3′. R: 5′-GCGCCCAATACGACCAAATC-3′.

RNU6 snRNA(U6) and GAPDH were used to be the internal control for *miR-144* and SMAD4. The relative quantitation of the value was determined using the 2^−ΔΔ*C*^_T_ calculation method and each sample was assayed in triplicate.

### Cell transfection

The cells (5 × 10^5^ per well) were seeded in six-well plates and transfected with purified *miR-144* mimics and control vector by using Lipofectamin 2000 (Invitrogen; Thermo Fisher Scientific, Inc.). Following transfection for 24 h, the next experiments were performed.

### Cell proliferation assay

Cell suspensions (1000 cells/well) were seeded in 96-well plates and were grouped according to the requirements. After 24 h, 10 μl (1g/l) of MTT (Beijing solarbio science & technology co., ltd.) were given to per well, then the cells were incubated 4 h at 37°C. Following discarding the supernatant, 100 μl of the DMSO were given to the two groups, respectively. The OD value was measured by 490 nm excitation to plot the proliferation curve using a microplate reader.

### Cell migration assay

We used wound scratch test for the cell migration assay. The *miR-144*-transfected SW620 cells were digested with trypsin, beat to single cell suspension using RPIM-1640 culture medium containing 1% FBS and counted using a hemocytometer plate. Then the cells were cultured in six-well (5 × 10^5^ A cells per well) and starved when they were integrated into monolayer cells. The same scratches were scored on each group of monolayer cells with 100 µl tip, and the cell mobility was calculated after 24 h of observation under a microscope.

### Cell invasion assay

24-well transwell invasion chamber of matrigel-coated transwell containing 8 m pores were used for the cell invasion. Cell (1 × 10^5^ cells) were seeded into the upper chamber in 100 µl serum-free MEM media and RPIM1640 culture solution containing 10% FBS were added to the lower chamber. The cells were fixed for 30 min in 4% paraformaldehyde and stained for 30 min with 0.1% hematoxylin 24 h later. After observing the cell staining with a microscope, the cell invasion rate was calculated.

### Luciferase reporter assays

When the cell (3.5 × 10^4^ cells per well, plated in 24-well plates overnight) density reaches about 70%, SW620 cells were cotransfected with SMAD4 plasmid (wild type/ mutant), *miR-144* mimics and anti-*miR-144* using Lipofectamine 2000.

### Western blotting analysis

After transfected with the corresponding plasmids by grouping, cells were lysed in Radio-Immunoprecipitation Assay buffer, centrifuged at low temperature (12,000 rpm/min, 20 min) and extracted from the protein supernatant. Protein concentration was detected using Bicinchoninic Acid Protein Assay Kit. The cell lysates (20 µg protein/lane) were separated by SDS-PAGE and then transferred onto PVDF membranes. After blocking with 5% non-fat dry milk for 1 h, the membranes were then incubated with Rabbit Polyclonal SMAD4 primary antibodies (cat. no. 10231-1-AP, 1: 1000 dilution) overnight at 4°C. Followed by washing, secondary antibodies (labeled with HRP) were incubated for 1 h at room temperature and detected with ECL Plus reagents. β-actin (cat. no. ab115777, 1: 1000 dilution) was used as the internal reference.

### Statistical analysis

The data were presented as mean ± S.D. Differences between two groups were assessed using a Student *t* test. A one-way ANOVA, followed by a least-significant difference test was used to compare differences amongst three or more groups. Statistical differences were considered significant at *P*<0.05. All statistical analyses were performed using SPSS 19.0 software.

## Results

### Expression of *miR-144* is decreased in human colon cancer tissues and colon cancer cell line

**Figure 1:**
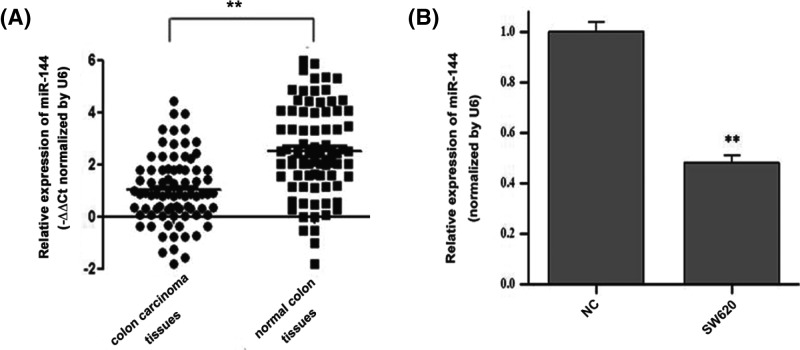
Expression of *miR-144* was detected in human colon tissues and colon cells (**A**) Relative expression levels of *miR-144* in colon tumors and normal colon tissues. (**B**) qRT-PCR analysis of *miR-144* levels in SW620 cells. Data are presented as the mean ± S.D. of three independent experiments. The expression level of *miR-144* was normalized to U6. **P<0.01.

The results showed that *miR-144* levels were significantly lower in colon tissues than in noncancerous colon tissues (Figure 1A). Furthermore, we found the expression levels of *miR-144* were also significantly lower in colon cell lines than in normal colonic epithelial cells (Figure 1B).

### *MiR-144* inhibits the proliferation of human colon cancer cells

**Figure 2:**
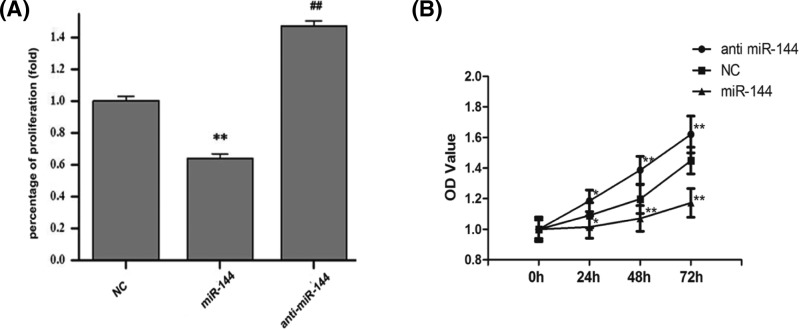
Effects of *miR-144* on the proliferation of human colon cancer SW620 cells were observed (**A**) Relative percentage of proliferation on SW620 cells in three groups. **P<0.01, for cells in transfected *miR-144* mimics group versus cells in NC group. ##*P*<0.01, for cells in *miR-144* inhibitor group versus cells in NC group. (**B**) OD value of SW620 cells in three groups. Data were obtained 0, 24, 48, and 72 h following transfection by MTT detection. **P<0.01, for cell proliferation repressed. Data are expressed as mean ± S.D.

The results showed that the proliferation of colon cancer cells was significantly inhibited in the transfected *miR-144* mimics group (*P*<0.01, Figure 2A), compared with the NC group. In addition, proliferation was significantly promoted in the anti-*miR-144* group, compared with the *miR-144* mimics group (*P*<0.01, Figure 2A). At the same time, we found that *miR-144* mimics repressed SW620 cells proliferation since 48 h after transfection (*P*<0.01, Figure 2B), which indicated that the *miR-144* could inhibit the proliferation of colon cancer SW620 cells.

### *MiR-144* inhibits the migration of human colon cancer cells

**Figure 3:**
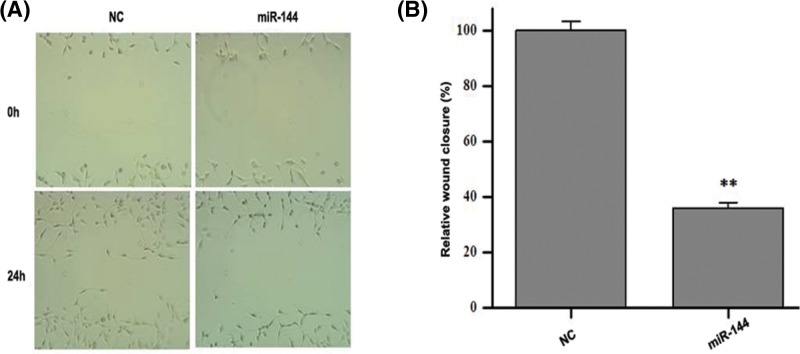
Effects of *miR-144* on the migration of human colon cancer SW620 cells were observed (**A**) Representative micrographs of cell migration assay using wound scratch test. (**B**) Data statistics of cell migration in wound scratch test. Data are expressed as mean ± S.D. **P<0.01.

Further scratch test showed that transfected *miR-144* mimics had an effect on colon cancer cells migration. As shown in Figure 3A, a significantly delayed wound closure of SW620 cells was observed in *miR-144* mimics-treated group compares with NC group. The quantitated results expressed in Figure 3B were also shown that the relative wound closure of SW620 cells was markedly decreased after *miR-144* mimics treatment (*P*<0.01). These results suggest that *miR-144* hold an inhibitory role in colon cancer cell migration.

### *MiR-144* inhibits the invasion of human colon cancer cells

**Figure 4:**
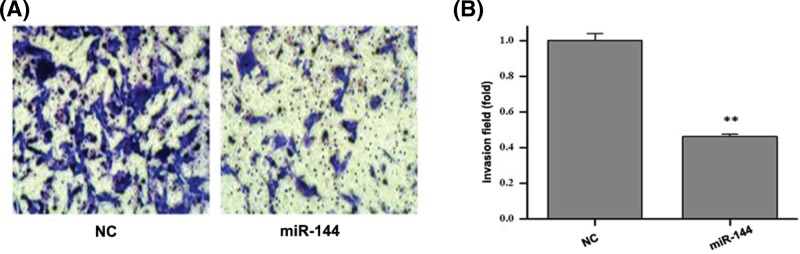
Effects of *miR-144* on the invasion of human colon cancer SW620 cells were observed (**A**) Representative micrographs of cell invasion assay using transwell chamber. (**B**) Data statistics of cell invasion in transwell chamber. Data are expressed as mean ± S.D. **P<0.01.

As shown in Figure 4, the invasion ability of colon cancer cells SW620 transfected with *miR-144* mimics was significantly lower than that of NC group (*P*<0.01), which indicated that the invasion ability of colon cancer cells treated with *miR-144* was inhibited.

### The target gene for *miR-144* may be SMAD4

**Figure 5:**
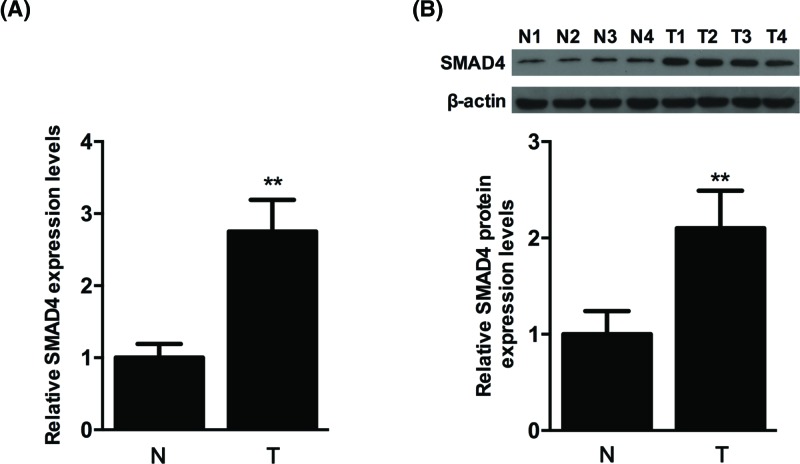
Expression of SMAD4 was detected in human colon tissues (**A**) mRNA levels of SMAD4 were detected in normal colon tissues and colon tumor tissues using qRT-PCR following *miR-144* transfection for 72 h. (**B**) Protein expression levels of SMAD4 were detected by using Western blotting analysis following *miR-144* transfection for 72 h. Data were normalized based on the levels of GAPDH. Each data point was obtained from three repeated experiments and expressed as the mean ± S.D. ** *P*<0.01.

**Figure 6:**
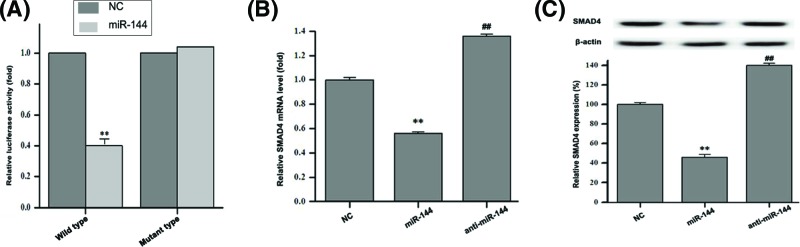
*MiR-144* negatively regulated SMAD4 in human colon cancer SW620 cells (**A**) SW620 cells were cotransfected with SMAD4 plasmid (wild type/mutant), *miR-144* mimics and anti-*miR-144*. **P<0.01, for the fluorescence intensity in wild-type cells versus the fluorescence intensity in mutant type cells. (**B**) Levels of SMAD4 mRNA were detected in the SW620 cells by using qRT-PCR following *miR-144* transfection for 72 h. **P<0.01, for mRNA level in *miR-144* mimics versus mRNA level in NC. ##*P*<0.01, for mRNA level in anti-*miR-144* mimics versus mRNA level in NC. Data were normalized based on the levels of GAPDH. (**C**) The expression levels of SMAD4 protein were evaluated in the SW620 cells by using Western blotting analysis following miR-144 transfection for 72 h. **P<0.01, for SMAD4 level in *miR-144* mimics versus SMAD4 level in NC. ##*P*<0.01, for SMAD4 level in anti-*miR-144* mimics versus SMAD4 level in NC. Data are expressed as mean ± S.D.

The above results indicated that *miR-144* inhibited SW620 cells proliferation, migration, and invasion. In order to examine the underlying mechanism, we examined the effects of *miR-144* on SMAD4 in the SW620 cells. First, the mRNA and protein levels of SMAD4 were detected by qRT-PCR and Western blotting in human colon cancer tissues. As seen in Figure 5, SMAD4, mRNA, and protein levels were significantly increased in colon tumor tissues compared with normal colon tissues (*P*<0.01). Based on this result, the luciferase activity of SMAD4 was identified. Luciferase reporter gene vector containing SMAD4-wild/mutant type 3′-UTR, *miR-144* mimics and anti-*miR-144* were cotransfected into wild-type and mutant SW620 cells. The results showed that the fluorescence intensity of *miR-144* mimics group in wild-type cells was decreased (*P*<0.01, Figure 6A), and it was unchanged in mutant type cells. This suggested that the target gene for *miR-144* may be SMAD4. After transfecting *miR-144* mimics and anti-*miR-144* mimics into SW620 cells respectively, we further detected mRNA levels and found that *miR-144* mimics reduced the transcription of SMAD4 (*P*<0.01, Figure 6B), conversely, anti-*miR-144* mimics increased the transcription level (*P*<0.01, Figure 6B), which demonstrated that *miR-144* might directly target SMAD4 in colon cancer cell lines. Furthermore, we found *miR-144* mimics repressed SMAD4 expression at protein level (Figure 6C, *P*<0.01). All the above results suggested that the target gene for *miR-144* may be SMAD4, by targetting which *miR-144* could inhibit SW620 cells proliferation, migration and invasion.

## Discussion

*MiR-144* has been extensively studied in other cancers such as gastric cancer [18], breast cancer [19], and lung cancer [20]; however, there is no report investigating it in colon cancer. In the present study, expression of *miR-144* is decreased in colon tissues and SW620 cells. We found that *miR-144* inhibited the proliferation, migration and invasion of human colon cancer cells by targetting SMAD4.

Numerous evidence strongly suggest that miRNAs play an important role in the pathogenesis of cancer [12,21,22]. Hence, we tried to explore the function of *miR-144* in colon cancer development and we found that expression of *miR-144* is decreased in colon tissues and cell lines. Not only in colon cancer, in osteosarcoma, the growth and invasion of cells are associated with the down-regulation of *miR-144* [23]. In thyroid cancer, the down-regulation of *miR-144* promotes cells invasion by targetting zinc finger E box [22]. In addition, *miR-144* suppresses proliferation, invasion, and migration in hepatocellular carcinoma [11]. Similarly, our studies also demonstrated that overexpressing *miR-144* inhibited colon cells proliferation, invasion, and migration.

Previous studies have shown that loss of SMAD4 is found in 30–40% colorectal cancer patients [1], which occurs late in adenoma-to-carcinoma sequence [16], leading to liver metastases, and these patients have a poor response to chemotherapy following with poor prognosis [17]. Onco-miRNAs play the role by down-regulating the targetting tumor suppressor expression in cancer progression [8,24]. In our study, we identified that *miR-144* could directly target SMAD4 in colon cells and cause its down-regulation. The mutation and down-regulation of SMAD4 contribute to many cancers progression. SMAD4 deletion spread metastatic squamous cell carcinomas and associated with lung metastasis and EMT in transgenic mice model [25]. The knockout of SMAD4 partially conferred BMP ligand resistance to the anti-growth effects in hepatocellular [26,27] carcinoma cells, which reduced the cells formation and migratory ability. In the present study, SMAD4 was considered to be a downstream target of *miR-144* in colon cancer progression. Our findings suggested that *miR-144* inhibited the onco-process of colon cancer cells through suppressing cell proliferation, migration, and invasion, which might be down-regulated by targetting SMAD4 expression.

In summary, we identified that *miR-144* inhibited colon cancer cells proliferation, invasion, and metastasis, which might be by targetting SMAD4. Direct at *miR-144*/SMAD4 pathway may provide a novel therapy for colon cancer patients. However, the present study has some limitations, such as additional cell lines representing different molecular subtypes and *in vivo* experiments should be further studied. As a next step, the prognostic potential of *miR-144* in the specific cohort of patients could also be investigated. We also know that K-ras or MSI status and p53 and APC are important for the targeted therapy of colon cancer development, but their correlation with *miR-144*/SMAD4 remains unclear, which needs further experimental demonstration.

## Abbreviations

GAPDH: glyceraldehyde-3-phosphate dehydrogenase
MiR: microRNA
NC: negative control
OD: optical density
qRT-PCR: quantitative real-time polymerase chain reaction
SMAD4: Mothers against decapentaplegic homolog4
